# 3D‐Printed Talus‐Calcaneus Prosthesis in Treating Ewing's Sarcoma: A Case Report

**DOI:** 10.1111/os.14279

**Published:** 2024-11-11

**Authors:** Weiyi Wang, Jingjing An, Minxun Lu, Xuanhong He, Zhuangzhuang Li, Yitian Wang, Taojun Gong, Yong Zhou, Li Min, Yi Luo, Chongqi Tu

**Affiliations:** ^1^ Department of Orthopedics, Orthopaedic Research Institute, West China Hospital Sichuan University Chengdu China; ^2^ Model Worker and Craftsman Talent Innovation Workshop of Sichuan Province Chengdu China; ^3^ Operating Room, Department of Anesthesiology, West China Hospital Sichuan University/West China School of Nursing, Sichuan University Chengdu China

**Keywords:** 3D‐printed, Ewing's sarcoma, prosthetic replacement, talus‐calcaneus

## Abstract

**Background:**

Malignant tumors originating in the talus are rare and present significant challenges in reconstruction. Traditional treatments, such as below‐knee amputation or tbiocalcaneal fusion, often result in significant loss of ankle function. After tumor resection, reconstruction of the talus and calcaneus is necessary to preserve ankle function. However, the intricate anatomical structure and unique location of the talus and calcaneus present significant challenges for prosthetic reconstruction.

**Case Presentation:**

Here, we present the case of an 11‐year‐old adolescent patient diagnosed with Ewing's sarcoma of the talus, accompanied by suspected involvement of the calcaneus. Following a comprehensive evaluation, a 3D‐printed talus‐calcaneus prosthesis, which is composed of a ultrahigh‐molecular weight polyethylene (UHMWPE) part and a titanium alloy part, was designed for talus and calcaneus reconstruction. In addition, a porous structure was designed to promote the integration of bone–prosthesis interface. The lesion was completely resected and the prosthesis was precisely installed. After 12 months follow‐up, patients demonstrated favorable function results with the Musculoskeletal Tumor Society (MSTS) score was 27/30, and the American Orthopedic Foot and Ankle Society (AOFAS) score was 92/100. The range of motion for dorsiflexion, plantarflexion, inversion, and eversion of the right ankle joint was measured as 10° and 35°, 15°, and 10°, respectively. The postoperative radiograph showed a good position of the prosthesis. No narrowed joint space was observed. Tomosynthesis shimadzu metal artifact reduction technology (T‐SMART) revealed that integration between bone and prosthesis was good.

**Conclusion:**

In this case, we present a case of 3D‐printed talus‐calcaneal prosthesis reconstructing talus and calcaneus. Favorable postoperative function outcome and good integration of the interface were observed. Therefore, this case provides an alternative therapeutic option for the treatment invasive talus tumor accompanied by suspicious contamination of the calcaneus. Nevertheless, a larger cohort study and with longer follow‐up is needed to evaluate the effectiveness and potential complications of this novel prosthesis.

## Graphical abstract

The reconstruction of the ankle is significantly challenged by the bone defect following talus‐calcaneus tumor resection. In this study, personalized talus‐calcaneus prosthesis was printed by 3D printing technology to reconstruct ankle and restore joint function of patient. During the follow‐up, the prosthesis and screws position were proper and the bone integration between prothesis and host bone were good.

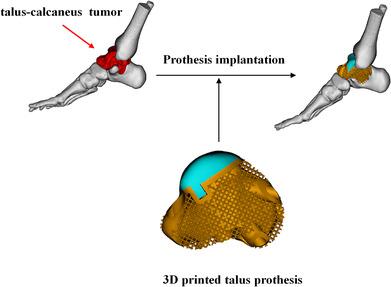

## 1 Introduction

Ewing's sarcoma (ES) is the second most prevalent primary malignant bone tumor, mainly involving long bones (47%), pelvis (26%), chest wall (16%), and spine (6%) [1, 2, 3, 4, 5, 6]. However, the hands and feet are rarely involved with the reported rate is about 3%–5% [7]. Furthermore, tumors originating in the talus are rare [8]. The standard treatment for malignant bone tumors of the foot is below‐knee amputation [9]. With advances in systemic therapies and surgical techniques, limb salvage surgery has emerged as a preferred alternative for treating malignant foot tumors, offering improved functional outcomes and quality of life [10]. Several methods have been reported to reconstruct talus or calcaneus, including tibiocalcaneal fusion [11], cryogenic autograft reconstruction [12], pedicled flap reconstruction [13], and prosthetic reconstruction [14]. Nevertheless, the above options were associated with complications such as ankle joint dysfunction and limb shortening, tumor recurrence, delayed bone healing, and long‐term postoperative immobilization, which limit the application of these limb salvage procedures [13, 14]. More importantly, though monostotic tumor such as talus or calcaneus tumor and related reconstruction strategy was reported, no case simultaneously invading the talus and calcaneus and effective limb salvage procedure was mentioned. Therefore, in patients with talus involvement and suspected contamination of the calcaneus, completely removing the tumor and recovering limb function is challenging.

Recently, three‐dimensional (3D) printing technology has been introduced for manufacturing customized implants in orthopedic surgery, offering advantages such as precise shape and size and a porous structure interface that can promote bone ingrowth [15]. To the best of our knowledge, this is no report of using a 3D‐printed prosthesis to treat a case affecting both the talus and calcaneus, simultaneously. In this case, we describe a case of ES with a rare anatomical presentation in the talus, including a suspected involvement of the calcaneus. A 3D‐printed prosthesis was applied to reconstruct the bone defect and restore the posterior foot anatomy and ankle function, providing a potential therapeutic strategy for the treatment of this rare and invasive talus tumor accompanied by suspicious contamination of the calcaneus.

## 2 Case Presentation

### 2.1 Clinical Data

An 11‐year‐old female patient presented at our hospital, reporting a 3‐month history of progressive pain in her right foot, experienced during both walking and rest. Before presenting to us, the patient had undergone a right talus incision biopsy in an upper first‐class hospital. The biopsy results revealed well‐differentiated bone trabeculae within the bone tissue, with well‐differentiated adipose tissue interspersed between the trabeculae.

**FIGURE 1:**
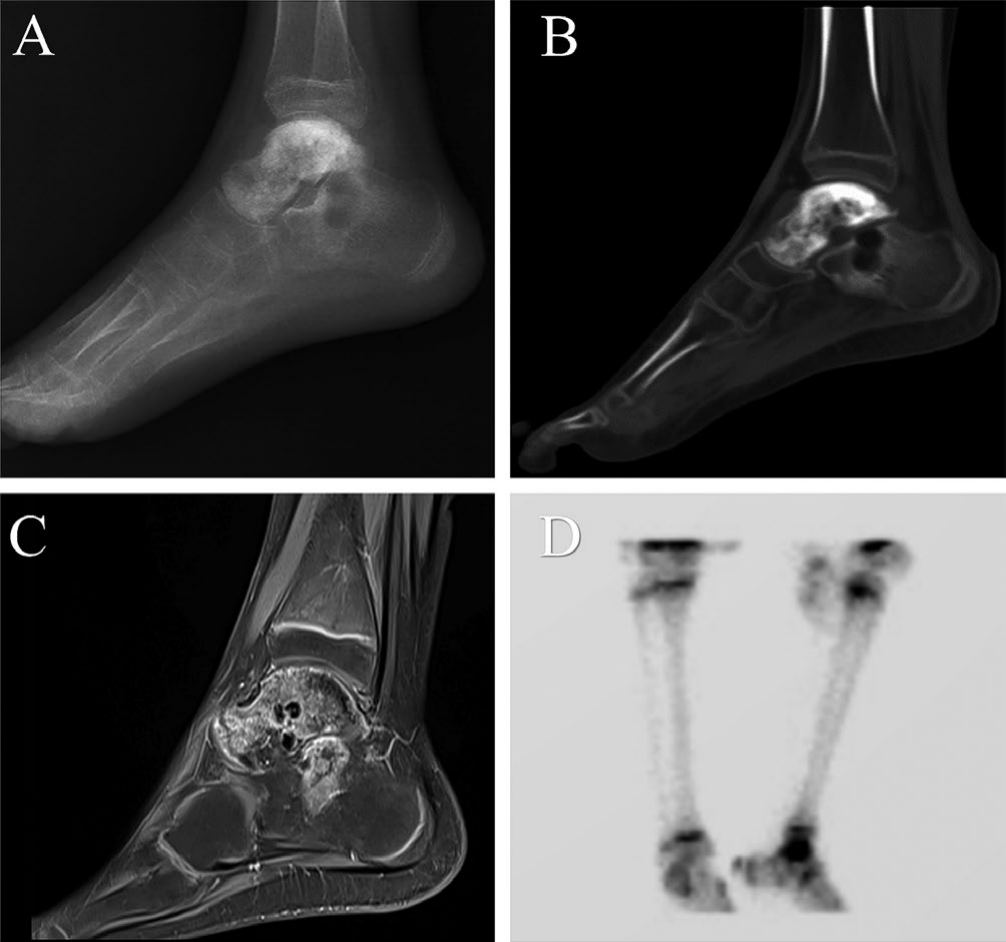
Preoperative radiograph (A), CT scan (B), MRI scan (C), and SPECT scan (D) of the patient with Ewing's sarcoma involving the talus, accompanied by suspected involvement of the calcaneus.

**FIGURE 2:**
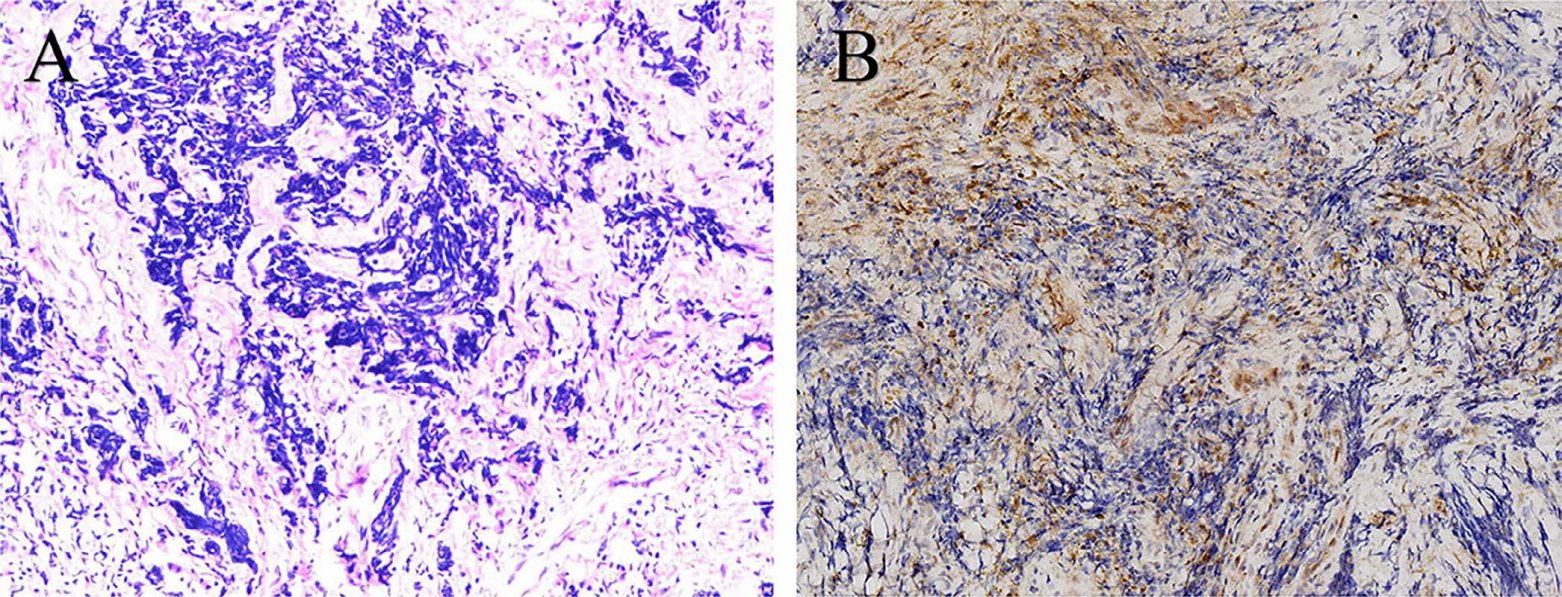
Histopathological images showed small cell malignant tumors. (A) hematoxylin and eosin (H&E) stain ×200 magnification; (B) immunohistochemistry (IHC) ×40 magnification.

The patient was admitted to our hospital for further diagnosis and treatment. The physical examination revealed swelling of the ankle, significant tenderness, and a limited range of motion. The MSTS score and the AOFAS score were 13/30 and 58/100 [16, 17], respectively. Dorsiflexion, plantar flexion, eversion, and inversion for the affected ankle were 10°, 30°, 15°, and 10°, respectively, while the contralateral ankle demonstrated values of 20°, 45°, 35°, and 30°, respectively. The radiograph revealed osteolytic bone destruction in the talus (Figure 1A). Computed tomography (CT) scan demonstrated a well‐defined osteolytic lesion in the talus and a local bone destruction in the calcaneus (Figure 1B). Magnetic resonance imaging (MRI) revealed a primary lesion in the talus and calcaneus (Figure 1C). The surrounding soft tissues of the ankle are intact without signs of invasion or cortical disruption. Single‐photon emission computed tomography (SPECT) showed the signal concentration around the right ankle joint (Figure 1D). Pathological biopsy of the right talus lesion suggests the diagnosis of Ewing sarcoma (Figure 2). Chest CT revealed no evidence of metastasis. The patient underwent 11 cycles of chemotherapy, and the pain and mobility issues in the right foot significantly improved after the treatment. The MRI indicates a reduction in the extent of the lesion edema. After a thorough preoperative evaluation, a 3D‐printed talus‐calcaneus prosthesis based on the patient's CT data was planned.

### 2.2 Design and Fabrication of the Prosthesis

**FIGURE 3:**
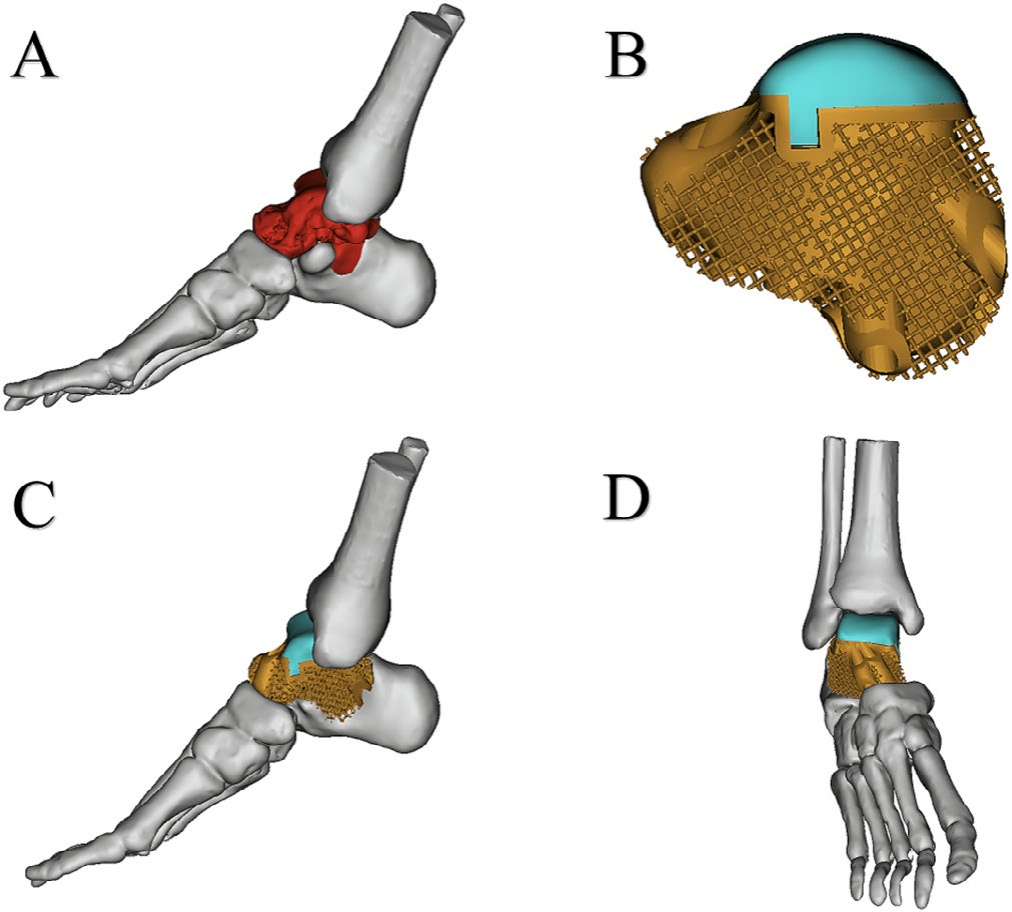
(A) Osteotomies were simulated on the virtual 3D models based on a combination of preoperative MRI and CT results; (B) the virtual 3D model of the 3D‐printed customized talus‐calcaneus prosthesis; (C) mediolateral view of the Simulating prosthetic replacement for the bone defect; (D) front view of the Simulating prosthetic replacement for the bone defect.

**FIGURE 4:**
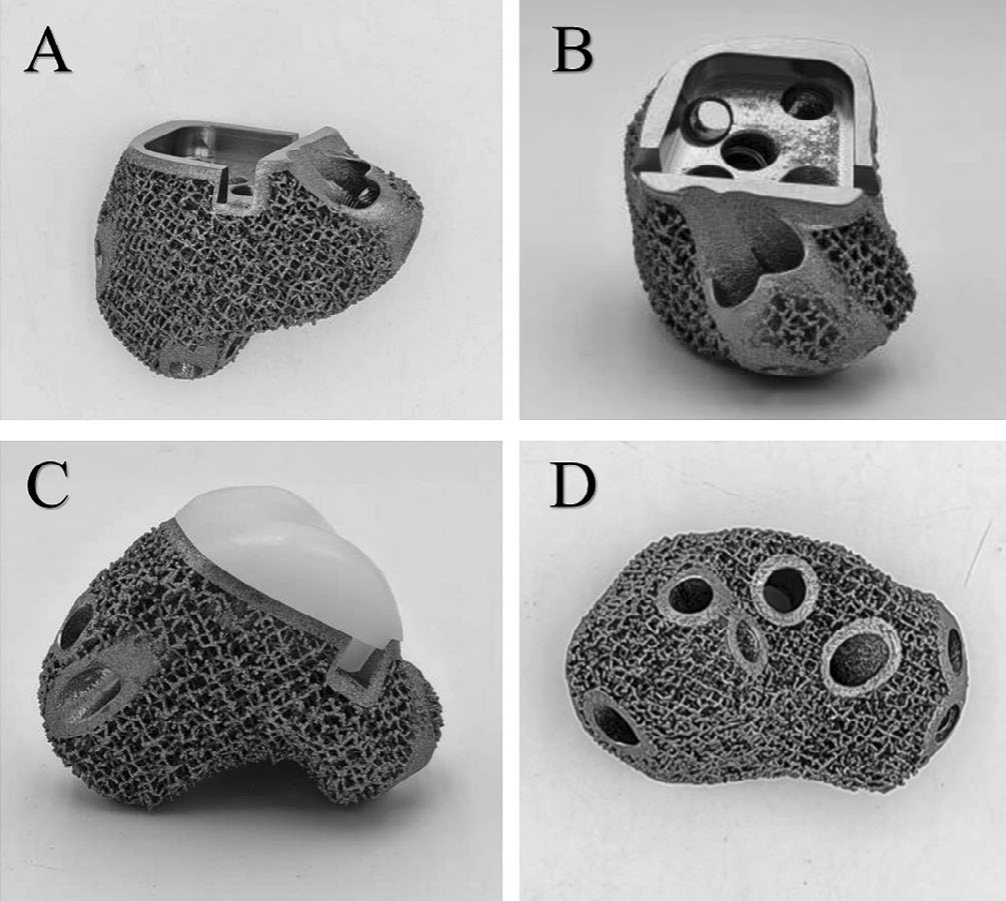
Physical photos of the 3D‐printed customized talus‐calcaneus prosthesis, (A) side view; (B) vertical view; (C) prosthetic segment component of ultrahigh‐molecular weight polyethylene (UHMWPE); (D) bottom view.

First, the patient's 3D CT data were imported into Mimics V20.0 software (Materialize Corp., Leuven, Belgium) to create virtual 3D models of the tumor and bone. Then the resection range was determined based on the extent of bone resection after evaluating the range from the MRI and the shape of the calcaneus and talus prosthesis were designed according to the shape of the bone defect (Figure 3A). Subsequently, the shape of the talus and calcaneus component of the prosthesis is generated by mirroring that of the contralateral side (Figure 3B,C). The prosthesis is composed of a UHMWPE part and a titanium alloy part, which are connected through a “snap‐fit” design (Figure 3B). The inner layer of the titanium alloy part was a solid titanium structure, while the outer layer was porous titanium with a pore size of 600 μm and an average porosity of 75%. The titanium alloy and UHMWPE connection includes predesigned screw holes for fixation of the prosthesis to the surrounding bone. Our clinical team designed the prosthesis, which was manufactured by Chunli Co. Ltd. (Tongzhou, Beijing, People's Republic of China), and fabricated by utilizing the electron beam melting technique (ARCAM Q10plus, Mölndal, Sweden), with Ti6Al4V powder as the printing material (Figure 4).

### 2.3 Surgical Technique

The surgery was executed by the senior surgeon (C.T.). Under general anesthesia, the talus and calcaneus were exposed through an anterior approach between the extensor hallucis longus tendon and the extensor digitorum longus tendon after applying a tourniquet and making a midline incision. The superficial peroneal nerve and anterior tibial artery were protected and the soft tissue was dissected to completely expose the talus and part of the calcaneus. The resection of the tumor was performed in two stages. The initial step involved the removal of the whole talus, followed by the excision of part of the calcaneus. During the operation, the polyethylene models were used as navigation (Figure S1) and the metal model was used for resection marking (Figure S2). In addition, high‐frequency electric knife was used to inactivate tumor wall along the outermost side of the polyethylene models. The surgical area was soaked alternately with a 10% povidone‐iodine solution, and the tumor wall tissue was cauterized with electrosurgical cauterization to further remove suspected lesions. The tumor in the calcaneus and talus was completely resected referring to the MRI.

**FIGURE 5:**
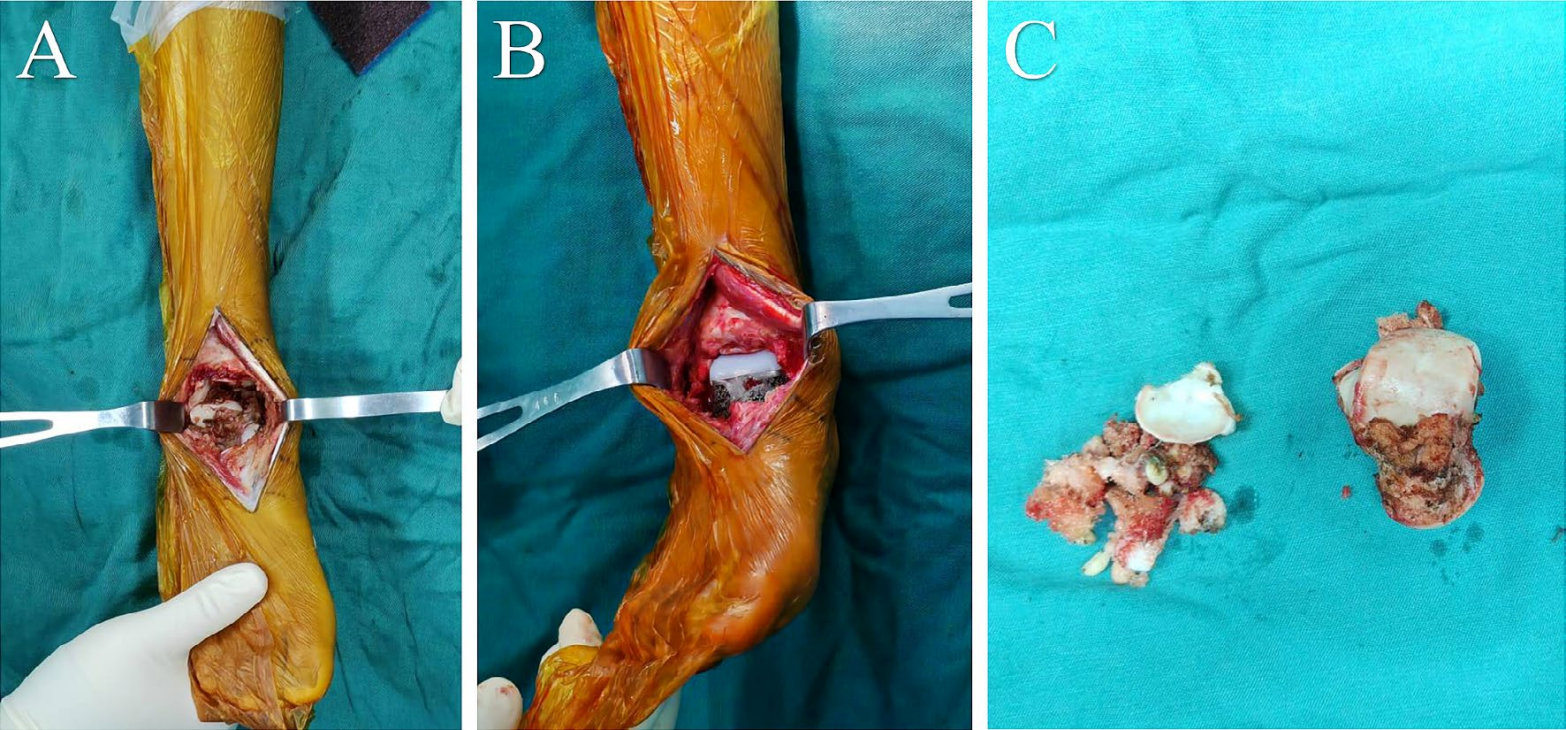
(A) The intraoperative pictures after the removal of the affected talus and calcaneus through anterior approach; (B) the modular prosthesis was inserted and fixed to the talus and calcaneus; (C) the resected talus and part of calcaneus.

Then, the residual calcaneus was shaped to a suitable bedplate. The metal modular components of the prosthesis were installed on the remaining calcaneus and navicular bones. The rotation angle was adjusted according to the preoperative design reference line. X‐ray repeatedly showed the internal and external rotation, varus, and valgus of the prosthesis and the insertion depth to a satisfactory position. Five screws and two medial wedge fusion screws were placed to fix the prosthesis. Then, the UHMWPE component was pressed and assembled onto the metal component (Figure 5). After confirming the positioning, the surgical site was thoroughly irrigated with saline, the incision was tightly sutured, and hemostasis was ensured. Free flaps of surrounding tissue fascia were used to adequately cover the wound, and a drainage tube was placed for drainage. The incision was layered sutured subcutaneously and the skin was tightly dressed.

### 2.4 Postoperative Management and Follow‐Up

**FIGURE 6:**
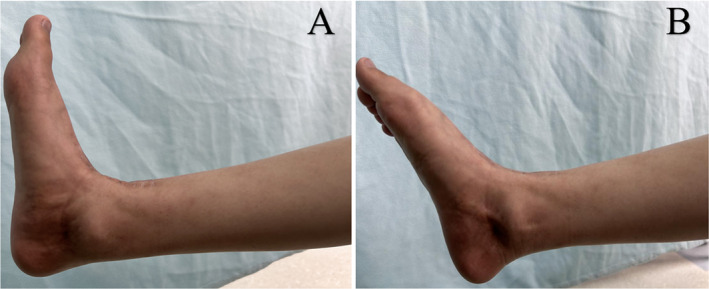
Postoperative 12 months ankle function, (A) dorsiflexion; (B) plantarflexion.

**FIGURE 7:**
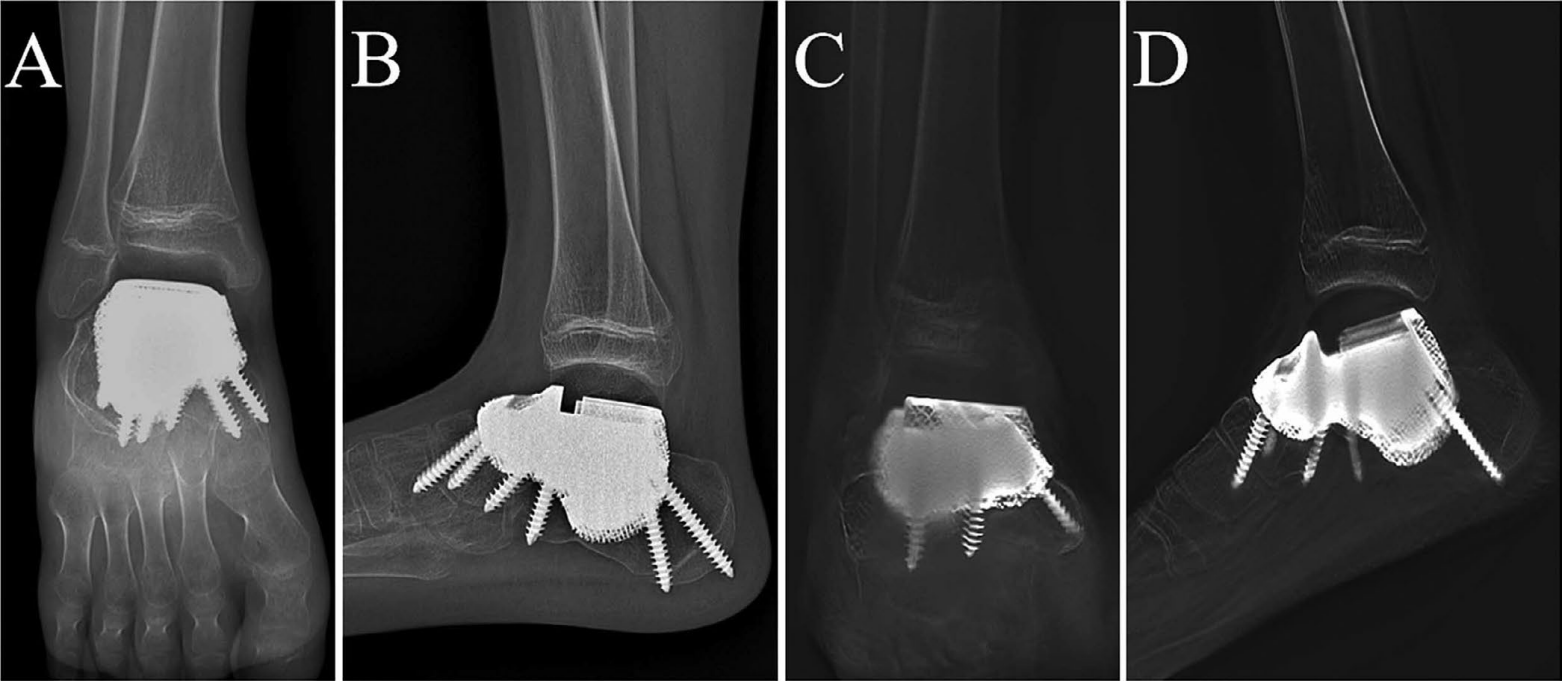
Postoperative 12 months X‐rays (A,B) and T‐SMART (C,D) indicated the prosthesis was well positioned.

Postoperatively, a plaster external fixator was applied to the right ankle joint to maintain dorsiflexion of the right foot. Initiation of range of motion training occurred at 2 weeks postoperatively, with gradual weight‐bearing activities commencing 4 weeks after the surgery. During the last follow‐up, which was 12 months postoperative, the patient had a satisfactory wound recovery and significant relief from pain. The range of motion for dorsiflexion, plantarflexion, inversion, and eversion of the right ankle joint was measured as 10° and 35°, 15°, and 10° degrees, respectively (Figure 6). The MSTS score was 27/30, and the AFOAS score was 92/100. During the 12 months follow‐up period, no complications related to the implant were observed. Radiograph showed a good position of the prosthesis and T‐SMART revealed an excellent integration of the prosthesis with the surrounding bone at the bone‐prosthesis interface (Figure 7).

## 3 Discussion

ES is an aggressive malignancy primarily affecting bones or adjacent soft tissues, predominantly in children and adolescents, peaking in the second decade of life [18]. It commonly arises in the long bones of limbs, pelvis [4]. The presence of ES in the foot, especially involving the talus, is exceedingly rare [19]. Currently, two primary surgical treatments exist for malignant foot tumors: amputation and limb salvage. Owing to the low complication rate and efficient local tumor control, below‐knee amputation has been considered the standard treatment for ES of the foot in the past [20]. However, this approach often results in considerable functional loss and diminished quality of life for the patient [2].

Owing to advancements in neoadjuvant chemotherapy and surgical techniques, limb salvage surgery presents a viable alternative for managing foot tumor, favored for its efficiency in enhancing patient satisfaction and preserving optimal ankle function [21]. Nevertheless, limb salvage surgery utilizing techniques such as tibiocalcaneal fusion [11], cryogenic autograft [12], pedicled flap [13], and prosthetic reconstruction [14]. These techniques encounter challenges including ankle dysfunction, limb shortening, tumor recurrence, reduced bone strength, delayed bone healing, and extensive postoperative immobilization. Those complications can constrain the practicality of these surgical methods [11, 12, 13, 14]. In 2008, Katagiri et al. [22] reported a case where arthrodesis with autogenous bone grafting and intramedullary nailing for a talar tumor. Despite the patient initially achieving an MSTS score of 90/100, the interface between the distal tibia and grafted iliac bone failed to union after 1 year. In 2014, Li and Wang [23] reported a case of limb salvage surgery using pedicled osteomyocutaneous fibular grafts. At the last follow‐up, there was no instances of local recurrence while one patient did experience a mild limp (Table 1).

**TABLE 1:** Literature review of reconstruction after resection malignant tumor of foot.

	No. of patients	Location	Reconstruction	Mean follow‐up (months)	Mean MSTS score (range)	Complications
Li et al. [23]	5	Calcaneus	Pedicled osteomyocutaneous fibular grafts	42.3	25.0 (−)	Two lung metastases, three fibula hypertrophies
Katagiri et al. [22]	1	Talus	Tibiocalcaneal fusion	47.0	27.0 (−)	One union, no local recurrence or distant metastasis
Sakayama et al. [12]	1	Talus	Frozen autograft	60.0	—	No local recurrence or distant metastasis
Gong et al. [25]	5	Talus	Endoprosthetic replacement	28.0	27.0 (25.0–29.0)	No prosthesis‐related complications occurred
Huang et al. [14]	1	Talus	Endoprosthetic replacement	24.0	27.9 (−)	No local recurrence or distant metastasis
Marco et al. [27]	4	Calcaneus	Iliac crest flap	156.0	29.0 (−)	Two screw breakage, one fracture
Antonio et al. [28]	1	Calcaneus	Endoprosthetic replacement	16.0	30.0 (−)	No local recurrence or distant metastasis

Recent advancements in 3D printing technology have facilitated the development of custom‐made prostheses, offering new possibilities for limb salvage [24, 25]. The use of a 3D‐printed talus‐calcaneus prosthesis for ES of the foot demonstrates the potential for personalized and functional limb reconstruction. This approach not only targets the tumor but also aims to restore the anatomical structure and function of the affected foot. Here, we describe the successful treatment of talus ES with a 3D‐printed prosthesis, which was precisely aligned with the surrounding joints and had predesigned holes that aided in the effective suturing of ligaments. In 2015, Imanishi and Choong [26] presented the first case of chondrosarcoma of the calcaneus treated with a 3D‐printed prosthesis. Postoperatively, the patient experienced a shorter recovery period. The AOFAS score for this patient was 82. At the last follow‐up, limited ankle motion was observed with 5° dorsiflexion, 25° plantar flexion, and 5° each of eversion and inversion. In contrast to our case, the patient achieved the MSTS score of 27/30 and the AOFAS score of 91/100. The range of motion for dorsiflexion, plantarflexion, inversion, and eversion of the right ankle joint was measured as 10° and 35°, 15°, and 10°, reflecting a good ankle function and an high postoperative satisfaction with the surgical outcome (Table 1). We believe that the patient to obtain such good results is caused by the following reasons. First, due to the modular design of our prosthesis supports tri‐directional screw fixation, leading to superior initial stability, enabling earlier commencement of functional exercises, and resulting in expedited patient recovery. Second, the 3D‐printed porous structure enhances bone integration, thereby achieving long‐term stability and decreasing the occurrence of long‐term complications. Third, to mitigate the risk of osteosclerosis, our study utilized a prosthesis with a modular configuration, fabricated from two disparate materials. The segment interfacing with the tibia was crafted from UHMWPE. Owing to UHMWPE's elastic modulus, which is comparatively low but closely approximates that of bone, the potential for wear is reduced.

In this case, there was no en bloc resection of the calcaneus. Given that the cartilage and ligaments between the calcaneus and the talus are well isolated from the tumor, we suspect that the calcaneus may have been contaminated during a biopsy at another medical institution. Therefore, we performed partial resection of the calcaneus. To ensure complete tumor removal via an anterior incision, achieve safe margin, and appropriately match the bone defect with the prosthesis, detailed preoperative planning was conducted. Based on x‐ray, MRI, CT, and SPECT, we determined the resection range and printed different models accordingly (Figure S1). During the operation, the polyethylene models were repeatedly used to check whether it matched the bone defect, so as to determine whether the tumor was completely removed and reached its safe margin. In addition, high‐frequency electric knife was used to inactivate tumor wall along the outermost side of the polyethylene models. In this process, the lesion was not only removed, but the potentially contaminated calcaneal lesion was also inactivated, achieving a safe margin. After surgery, the patient underwent 33 courses of radiotherapy targeting the affected area (Figure S3), each course delivering a dose of 200 cGy. There were no signs of tumor recurrence.

To conclude, we document an innovative limb salvage surgical technique for ES of the talus accompanied by suspicious contamination of the calcaneus, employing a 3D‐printed prosthesis for the talus‐calcaneus replacement. This approach not only targets the tumor but also aims to restore the anatomical structure and function of the affected foot. Following surgery and adjuvant treatment, the patient achieved optimal function and expressed satisfaction with the clinical outcomes. Furthermore, no signs of recurrence or complications were observed.

## 4 Conclusion

In this case, we present a case of 3D‐printed talus‐calcaneal prosthesis reconstructing talus and calcaneus. Favorable postoperative function outcome and good integration of the interface were observed. Therefore, this case provides an alternative therapeutic option for the treatment invasive talus tumor accompanied by suspicious contamination of the calcaneus. Nevertheless, a larger cohort study and with longer follow‐up is needed to evaluate the effectiveness and potential complications of this novel prosthesis.

## Author Contributions

W.W., J.A., and C.T. contributed to the conceptualization and design of this article. M.L., Z.L., and T.G. were involved with the acquisition of the subject and data. L.M., Z.L., and C.T. were involved in the design of the prosthesis. Y.W., X.H., Y.L., Y.Z., and C.T. participated in the evaluation of the patient after surgery. All authors contributed to the article and approved the submitted version.

## Ethics Statement

This study was conducted in accordance with the revised 2008 Declaration of Helsinki and received approval from the Ethics Committee of West China Hospital.

## Consent

The patient provided written consent via the informed consent form prior to the surgical procedure.

## Conflicts of Interest

The authors declare no conflicts of interest.

## Supporting information


**Figure S1:** (A) Polyethylene models of different specifications. (B) In vitro demonstration of polyethylene model navigation partial calcaneal resection.
**Figure S2:** (A–C) The metal model is marked on the calcaneus. (D–F) In vitro demonstration of the calcaneus marked with a metal model from anterior incision.
**Figure S3:** Radiotherapy target area. (A) Side view; (B) Front view.
