# Supplementary material for: 3D‐Printed Talus‐Calcaneus Prosthesis in Treating Ewing's Sarcoma: A Case Report
Source: Orthop Surg. 2024 Nov 11;17(1):288–94. doi: 10.1111/os.14279 (PMC11735376; doi:10.1111/os.14279)
Supplement: Supplementary file 1 — Figure S1: (A) Polyethylene models of different specifications. (B) In vitro demonstration of polyethylene model navigation partial calcaneal resection. Figure S2: (A–C) The metal model is marked on the calcaneus. (D–F) In vitro demonstration of the calcaneus marked with a metal model from anterior incision. Figure S3: Radiotherapy target area. (A) Side view; (B) Front view. [file OS-17-288-s001.docx]

**Supplementary Materials**


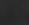
**
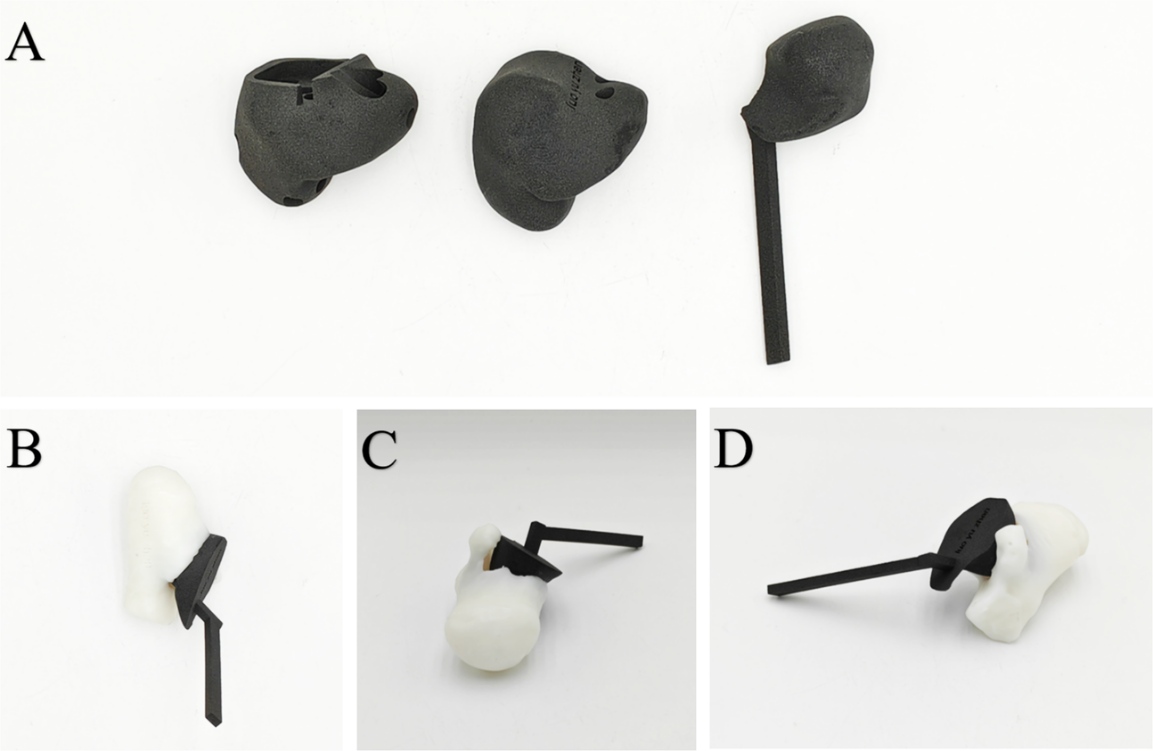
**

**Figure S1**. (**A**) Polyethylene models of different specifications. (**B**) In vitro demonstration of polyethylene model navigation partial calcaneal resection.


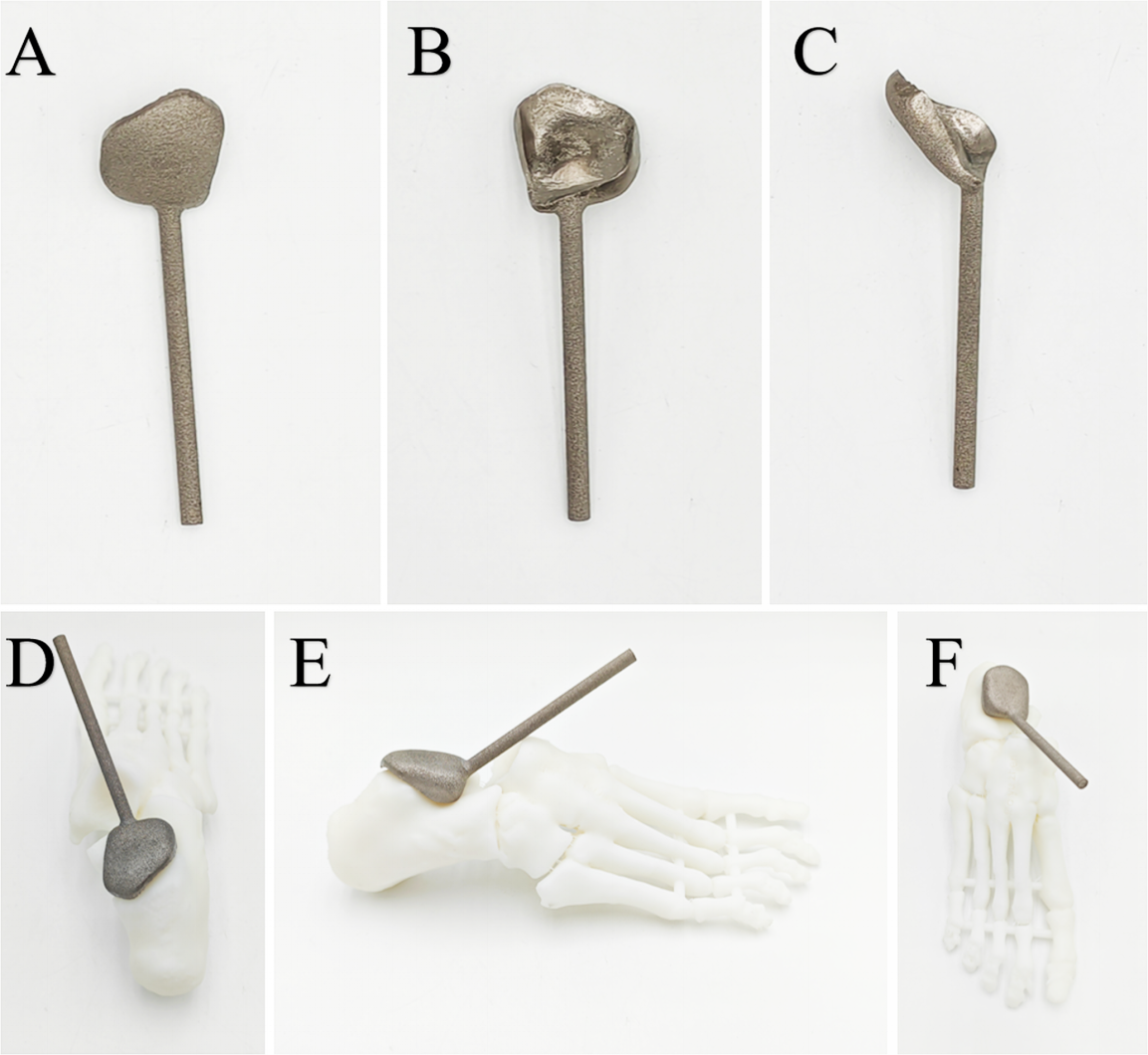


**Figure S2**. (**A-C**) The metal model marked on the calcaneus. (**D-F**) In vitro demonstration of the calcaneus marked with a metal model from anterior incision.

**
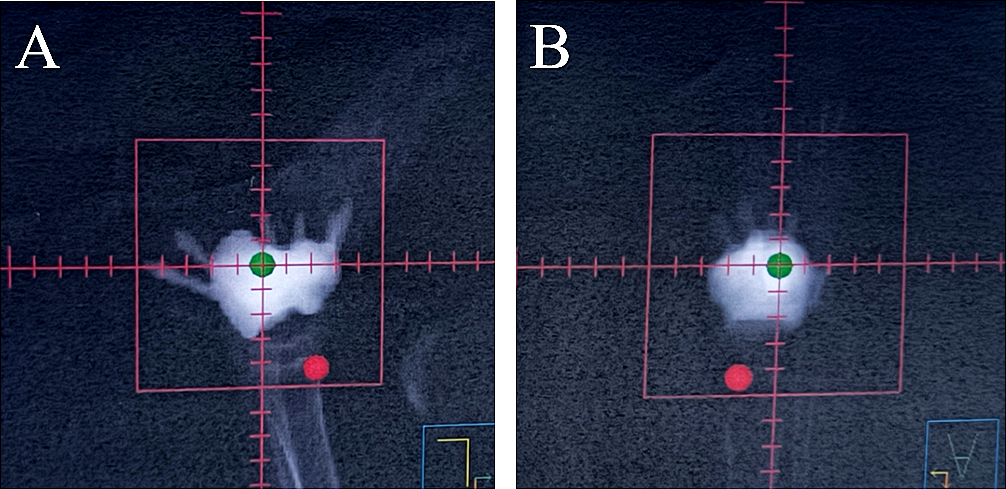
**

**Figure S3**. Radiotherapy target area. (**A**) Side view; (**B**) Front view.
